# Effects of a 4‐Month Supervised Aerobic‐Strength Training on Cognitive Functions and Clinical State of Patients with Parkinson’s Disease

**DOI:** 10.1002/alz.089825

**Published:** 2025-01-09

**Authors:** Jozef Ukropec, Lucia Slobodová, Karin Marček Malenovská, Ali Amiri, Michaela Konečná, Igor Straka, Radka Klepochová, Zuzana Košutzká, Martin Krššák, Peter Valkovič, Barbara Ukropcová

**Affiliations:** ^1^ Biomedical Research Center, Slovak Academy of Sciences, Bratislava Slovakia; ^2^ Institute of Pathophysiology, Faculty of Medicine, Comenius University, Bratislava Slovakia; ^3^ University Hospital Bratislava and Faculty of Medicine Comenius University, Bratislava Slovakia; ^4^ High‐Field MR Centre, Medical University of Vienna, Vienna Austria; ^5^ Institute of Normal and Pathological Physiology, Center of Experimental Medicine, Slovak Academy of Sciences, Bratislava Slovakia

## Abstract

**Background:**

Physical exercise improves clinical state of patients with Parkinson’s disease (PD), and evidence from experimental models suggests it has a potential to slow down the disease progression. Improved glucose metabolism as well as exerkines, bioactive molecules released into circulation with each exercise bout, contribute to the synchronized exercise‐induced adaptive response at a systemic level. Our aim was to assess effects of exercise on clinical state and molecular changes in cerebrospinal fluid (CSF) and blood of patients with PD.

**Methods:**

Patients with PD (H&Y score I‐III, 9F/17M; age 63.3 ± 8.4yrs; BMI 26.5 ± 5.9kg/m^2^) underwent supervised 4‐month aerobic‐strength training (3×1h/week). Clinical state (MDS‐UPDRS), body composition (BIA/MRI), cognitive functions (CogState, ACE‐R, TMT‐A&B, DSST, RAVL test), VO_2_max (Rockport 1 mile walk test), muscle strength (dynamometry), resting metabolic rate, metabolic substrate flexibility (indirect calorimetry), and insulin sensitivity (euglycemic hyperinsulinemic clamp) were assessed and CSF was sampled by atraumatic lumbar puncture (in a subpopulation) before / after 4‐month intervention. Extracellular vesicles (EVs) were isolated from serum and CSF by size exclusion chromatography (SEC). Proteomic analysis (mass spectrometry) of the samples is currently ongoing.

**Results:**

Four‐month aerobic‐strength exercise improved or tended to improve clinical state (MDS‐UPDRS, p<0.05), body composition (reduced: BMI, p = 0.007; body fat, p = 0.08; visceral fat, p = 0.038) and cognitive performance (ACE‐R verbal production subscore, p = 0.029; psychomotor function, p = 0.04; visual learning & short‐term memory, p = 0.009) in patients with PD. Training reduced circulating HbA1C, improved whole‐body metabolic flexibility (substrate preference based on the substrate availability, the hallmark of healthy metabolism) and muscle strength (all p<0,05). Proteomic analysis of the samples obtained before and after training is in progress.

**Conclusions:**

A relatively short aerobic‐strength exercise has favorable effects on cognition and clinical state in patients with PD which could be, at least in part, related to the improvements in patients’ physical fitness and metabolism. It is plausible to speculate that the specific training‐induced changes in the proteome of CSF, blood and EVs could be involved in the adaptive response underlying the exercise‐induced health benefits in PD.

**Funding**: APVV‐20‐0466, VEGA‐2/0076/22, ADDIT‐CE Horizon Europe 101087124

